# Synthetic biology for evolutionary engineering: from perturbation of genotype to acquisition of desired phenotype

**DOI:** 10.1186/s13068-019-1460-5

**Published:** 2019-05-09

**Authors:** Jina Yang, Beomhee Kim, Gi Yeon Kim, Gyoo Yeol Jung, Sang Woo Seo

**Affiliations:** 1 School of Chemical and Biological Engineering, Seoul National University, 1 Gwanak-ro, Gwanak-gu, Seoul, 08826 South Korea; 2 Institute of Chemical Process, Seoul National University, 1 Gwanak-ro, Gwanak-gu, Seoul, 08826 South Korea; 3 Interdisciplinary Program in Bioengineering, Seoul National University, 1 Gwanak-ro, Gwanak-gu, Seoul, 08826 South Korea; 4 Department of Chemical Engineering and School of Interdisciplinary Bioscience and Bioengineering, Pohang University of Science and Technology, 77 Cheongam-ro, Nam-gu, Pohang, Gyeongbuk 37673 South Korea

**Keywords:** Synthetic biology, Evolutionary engineering, Biosensor, Cell factory, Directed evolution

## Abstract

With the increased attention on bio-based industry, demands for techniques that enable fast and effective strain improvement have been dramatically increased. Evolutionary engineering, which is less dependent on biological information, has been applied to strain improvement. Currently, synthetic biology has made great innovations in evolutionary engineering, particularly in the development of synthetic tools for phenotypic perturbation. Furthermore, discovering biological parts with regulatory roles and devising novel genetic circuits have promoted high-throughput screening and selection. In this review, we first briefly explain basics of synthetic biology tools for mutagenesis and screening of improved variants, and then describe how these strategies have been improved and applied to phenotypic engineering. Evolutionary engineering using advanced synthetic biology tools will enable further innovation in phenotypic engineering through the development of novel genetic parts and assembly into well-designed logic circuits that perform complex tasks.

## Background

Synthetic biology aims to create or redesign new biological systems to achieve specific purposes. Comparing with traditional bioengineering, synthetic biology is based on the prediction and design. Evolutionary engineering focuses on obtaining desired functions of a system through overcoming a lack of information. Especially, the complex functions required for industrial strains such as optimized production pathway, tolerance of product, and genomic stability are difficult to gain with rationally assigned modifications only. Therefore, generating massively diverse phenotypes and screening improved variants out of them can circumvent the limitation. Evolutionary engineering mimics Darwinian selection, and the beneficial phenotypes are propagated to offspring. Thus, the addition of appropriate selection pressure is a key factor to the acquisition of the desired phenotype.

In this aspect, synthetic biology can provide tools to accumulate genetic mutations and to link or convert these genotypes into detectable phenotypes. Recently, techniques for phenotypic perturbation such as transcription/translation machinery engineering, CRISPRi/a, sRNA, MAGE derived techniques, error-prone replication machinery, and genome shuffling were demonstrated [1–12]. In addition, phenotypic-specific biosensor/selector, biomolecule compartmentalization technique, and obtaining desired function based on phage progeny were developed. To provide further understanding and insight, recently developed techniques of synthetic biology used to evolve microorganisms to achieve the desired phenotype will be summarized in this review.

### Phenotypic perturbation

Rewiring protein expression or modifying specific activity of a protein causes phenotypic perturbation. In general, cellular traits which are required to be utilized in the industry are difficult to be shown because flux redirection and biochemical accumulation in a cell decrease cellular fitness. High-throughput, intensive, multiplex genetic mutations can expand the phenotype spaces enough to bring desired phenotype.

#### Transcriptional regulation perturbation

**Fig. 1:**
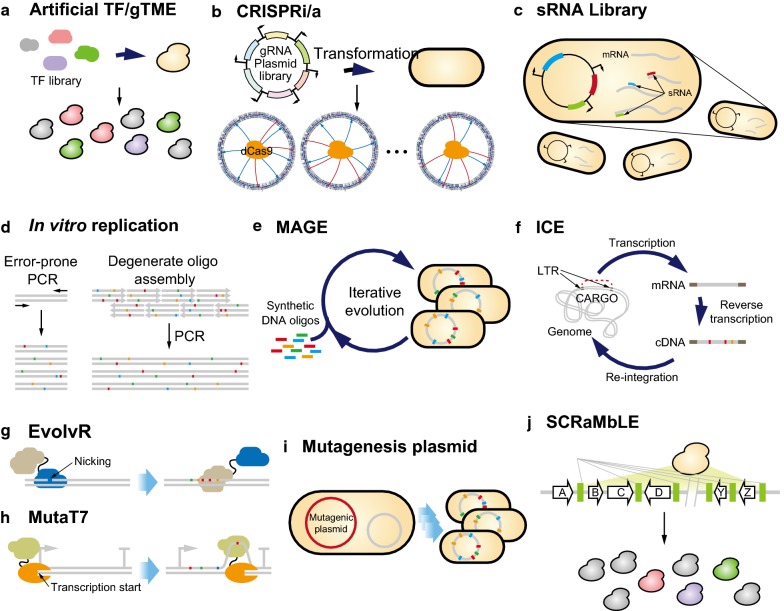
Phenotypic perturbation methods. **a**, **b** Represent methods to alter transcriptional regulation. **a** Artificial TF and gTME library; transformation with artificial transcription factor or of global transcription machinery libraries that are generated by error-prone PCR results in phenotypic perturbation by unpredictable regulation changes. **b** CRISPRi/a; transformation with guide RNA (gRNA) plasmid array and dCas9 repressor/activator results in alter cellular regulations. **c** Perturbation of translational expression level through binding of sRNA and RNA binding protein. Expression of multiple sRNAs can repress multi-gene expression. **d** Representative two method for in vitro mutagenesis. A error-prone PCR introduce mutations during PCR and PCR with oligos harboring degenerate sequence diversify DNA sequence after being assembled. **e** MAGE technique introduces multiple mutations in genome through automation of iterative synthetic oligo recombineering. **f** ICE generate mutated DNA in vivo. A designed cassette composed of transposable element and target gene (CARGO) is transcribed and then reverse transcribed in an error-prone manner. The resulting cDNA is integrated back into the chromosome. **d**, **g** are methods to introduce mutations on target region. **g** EvolvR consists of a DNA polymerase fused with nCas9 which is recruited by gRNA into target region. After a DNA is nicked by the nCas9, the error-prone DNA polymerase performs error-prone strand displacement synthesis. **h** MutaT7 consists of a cytidine deaminase fused to T7 RNA polymerase. It is recruited to T7 promoter and mutations are loaded during transcription before transcription is terminated by terminator. **i** A plasmid carrying proof reading deficient DnaQ and factors conferring replication fidelity generates mutations during cell growth. **j** The pre-positioned loxPsym sites are recombined, inverted, and deleted by Cre induction and results various phenotypes

Synthetic biology techniques can provide synthetic transcription factors to elicit de novo regulation. One of the examples is an artificial transcription factor which has new regulatory functions in a cell. Park et al. constructed artificial transcription factor (TF) library capable of random regulation of endosomes gene expression (Fig. 1a) [9]. The library contained more than 100,000 artificial transcription factors, each of which consisted of zinc finger domains with different binding specificities and transcriptional activator or repressor domain. The artificial transcription factors could bind endogenous DNA randomly, so when it binds a specific locus with regulatory role, cellular metabolic network would be perturbed.

Cells regulate the metabolic network with multiple regulation factors to suit specific environments and cellular state. Rewiring the innate regulation leads cells to new phenotypes. Alper et al. aimed to change interactions between host transcription factors (TF) and their targets (Fig. 1a) [1]. The evolutional potent of global transcription machinery was demonstrated by global transcription machinery engineering (gTME). One of the transcription machineries, TATA-binding protein encoded in *SPT15* was chosen to generate library by error-prone PCR. The library was cultured under high ethanol concentration as selection pressure, and tolerant variants were selected. The effect of combined mutations on host global transcription factors brought differential gene expressions of hundreds of genes compared to wild-type cells, and these simultaneous alterations of multigene expression elicited improved phenotype. The gTME was employed for mutagenesis of both model strain (*Saccharomyces cerevisiae*) and non-model strain (*Yarrowia lipolytica*). A *spt15* variant which brought ethanol-tolerant phenotype to *S. cerevisiae* was screened, and the effect on physiology was analyzed. The screened *spt15* variant was more resistant to osmotic shock, and growth inhibition was smaller than that of wild-type *SPT15* when glucose was fed at a high concentration [13]. This is a great advantage in the fed-batch culture, but the changes in metabolic regulations still have to be analyzed. *S. cerevisiae* which is more tolerant to crude substrates, corn cob acid hydrolysates, and metabolizes xylose was screened from *spt15* library [14]. Transcriptome, metabolic flux analysis, and phenotyping were performed by Wadhwa et al. on the *spt15* mutant screened from their previous study. They found that the *spt15* mutant affected phosphate limitation, which rewired central carbon metabolism and increased flux to the isoprenoid pathway [15, 16]. Moreover, the applicability of gTME was demonstrated in *Y. lipolytica* to modulate phenotype by expressing additional *Yl*-*spt15* variants without deleting original transcription factor. The accumulation of fatty acids and lipid bodies was influenced by the gene expression ratio of the wild-type *SPT15* and *Yl*-spt15 variants as well as the mutations on *Yl*-*SPT15* [17]. There are also several successful studies applying gTME to improve *Escherichia coli* phenotypes such as high hyaluronic acid production and organic-solvent tolerance [1, 18]. In these studies, mutant libraries of major sigma factors, *E. coli rpoD* and/or *rpoS*, were screened under appropriate selection pressure.

In addition to the studies based on the host TF, an exogenous transcriptional regulator was employed for gTME. The regulators of a radio-resistant bacterium, *Deinococcus radiodurans*, have often been used to bring diverse tolerance in *E. coli*. The tolerance of *E. coli* to multiple stress was increased by introducing one of the global regulators, IrrE or response regulator, DR1558 from *D. radiodurans* [19, 20]. Although transcriptome and proteome of ethanol-tolerant strain screened from *irrE* mutant library have been altered, the exact mechanism that gives tolerance remained to be unveiled [5]. Artificial TFs and gTME technique usually change expression level of a tremendous number of genes in unpredictable mechanism. To traverse more guided phenotype space, targeted cellular reprogramming is also regarded as an efficient strategy to generate desired phenotype. One of the traditional methods is to generate combinatorial library by replacing promoters of target genes to other synthetic promoters with different strengths. Blazeck et al. selected genes involved in lipogenesis, and the overexpression or deletion of these target genes showed different amounts of lipid accumulation [21]. Although they succeeded in improving strains to increase the total lipid production by 60 times, there are still some limitations to search large phenotype spaces due to low-efficiency and laborious recombination steps.

A nuclease-deficient Cas9 protein-based transcriptional interference/activation system, CRISPRi/a, made it possible to modulate expression level of target genes without replacing their promoters (Fig. 1b) [10]. Using both dCas9-repressor and dCas9-activator, Deaner et al. enabled regulation of target gene expression in graded manner within a wide range based on the distance between a target location and a core promoter, which affects the regulation fold-change. They applied CRISPRi/a system to systematically test enzyme perturbation sensitivities (STEPS), and rapidly improved glycerol and 3-dehydroshikimate (3-DHS) production in yeast [22]. However, a dCas9-repressor and a dCas9-activator share their gRNAs, which limits their ability to program the expression levels of multiple genes in a cell. To overcome this limitation, it was examined whether the dCas9-activator could also role as a repressor depending on the binding location. Accompanied with a ribozyme-sgRNA array, bifunctional role of the dCas9-activator enhanced multiplexing power of CRISPRi/a in yeast [23].

CRISPRi/a techniques have also been adopted to optimize expression levels of multiple endogenous genes in prokaryotes (Fig. 1b). Wang et al. screened high-lycopene-producing-*E. coli* among knockdown library targeting 56 phosphatase-encoding genes which were systematically identified [24]. Using combinations of sgRNAs which were targeting different genes and different locations, Wu et al. dynamically optimized expression levels of three genes in the competing pathways for the production of *N*-acetylglucosamine in *Bacillus subtilis* [25]. Bikard et al. first demonstrated that the fusion of the ω-factor of *E. coli* RNAP, encoded in *rpoZ*, to dCas9 enabled implementing CRISPRa system in prokaryote [3]. Later, Dong et al. developed an improved version of CRISPRa system for prokaryote. They utilized gRNA incorporating MS2 aptamer (scRNA) which recruited SoxS activator fused with MS2 coat protein (MCP-SoxS) to the target region; so, miss-folding risk from fusion of dCas9 with large protein domain was eliminated [7]. With the information, it is possible to construct the library based on these CRISPRi/a systems to search phenotypic space, and the size of the space is depending on the variability of the guide sequences of sgRNAs. Recently, *E. coli* genome-scale guide sequence library has been developed and used to screen the genes conferring toxic chemical tolerances [26]. Accompanied with the genome-scale guide sequence library, advanced gene assembly methods allow the CRISPRi/a system to be one of the most efficient tools to generate phenotypic perturbations [27–29]: CRISPathBrick (*E. coli* up to 5 gRNAs), CRISPR-LEGO (*S. cerevisiae* up to 5 gRNAs), and ASAP-cloning (mammalian cell up to 9 gRNAs).

#### Translational regulation perturbation

To explore more diverse phenotypes with less laborious experiments, a library should be small in size but cover wide range of expression levels. Based on the predictive design tools for 5′untranslated region (5′UTR) [30, 31], algorithms were developed to generate the degenerate 5′UTR sequence covering a defined range of translation initiation rate (TIR) [32, 33]. Moreover, a RedLibs algorithm was proven to generate smallest library with uniformly discrete TIRs [34]. RedLibs reduced the risk and effort of the analysis step by minimizing the size of library through leaving the sequences meaningful to investigate only. The aforementioned methods generate a library resulting a subtle sequence change in the relatively narrow region such as ribosome-binding sites (RBSs). Due to a small change in DNA sequence, these mutations can be restored by DNA mismatch repair (MMR) system during replication in bacteria. The adverse effect of MMR system on the library generation had been examined, and removal of MMR system increased recombination efficiency using single-stranded DNA (ssDNA) [12, 35]. On the other hand, removal of MMR system resulted in an unintended increase in background mutation rate. Using ssDNA containing chemically modified bases could be an alternative strategy to improve oligo-mediated recombination [36]. Recently, genome-library-optimized-sequences (GLOS) design rule enabled sufficient recombineering in MMR-proficient strain with ssDNA by remaining only sequence variants carrying more than 6 base pairs (bp) mismatch at the targeted region [37]. Despite the development of recombineering-based library generation strategies, genome-scale multiplexing engineering is still limited.

Na et al. demonstrated that rationally designed synthetic small regulatory RNA (sRNA) could be served as a tool to control translation efficiency of multiple target genes simultaneously for genome-wide screening of the desired phenotype (Fig. 1c) [8]. Noh et al. advanced sRNA-mediated regulation system. To precisely tune target gene expression, they modulated a content of sRNA in a cell [38]. To fine-tune the level of multiple gene expression, a larger size comparing to a knock-down library with the same number of targets is required. A novel method using predetermined orthogonal sRNA-target sequence pool enabled balancing the expression levels of multiple genes composing a synthetic pathway. By assembling the pathway with gene cassette carrying a sRNA-target sequence around the TIR of each gene, the gene expression levels could be diversified with pre-built sRNA library that consisted of several sRNAs with different transcription levels [39]. With this method, the designed beta-Carotene production pathway was efficiently optimized using reusable sRNA pool.

An orthogonal translation system is a fundamental requirement to reprogram cellular gene regulation by incorporation of non-natural amino acids in target protein or expand genetic code. Researchers attempted to develop orthogonal ribosome and orthogonal aminoacyl tRNA synthetase/tRNA set. The nature of a ribosome consisting of two subunits makes it difficult to modify ribosome functionality. Although a mutant 30S subunit with changed elongation property and modified 16S rRNA to recognize alternative Shine–Dalgarno sequence was constructed, the engineering 50S subunit was limited because of the interference in orthogonal subsets. To circumvent these limitations, a tethered ribosome (Ribo-T) and stapled orthogonal ribosome where the large and small subunit rRNAs are linked were demonstrated [40, 41].

#### Directed evolution of CDS/specific locus/plasmid

Evolutionary engineering of protein or plasmid is one of the key methods to improve protein activity and cellular phenotype. Furthermore, intensive protein-directed evolution could create superior functionality such as solvent tolerance and non-natural function [42]. An error-prone PCR or assembly methods with DNA fragments harboring degenerate sequences are generally used to create mutated proteins in a broad region or a specific position, respectively (Fig. 1d). Usually these methods are accompanied with transformation and in vivo selection step.

Multiplex automated genome engineering (MAGE) demonstrated that combinatorial sequence changes could be introduced at multiple loci in *E. coli* genome (Fig. 1e) [12]. Using synthetic 90-mer oligo pools containing degenerate sequences, a simple automated cycle consisting of three steps (cell growth and recovery, making recombination-competent cell, and electroporation step) was repeated to generate genetic diversity. However, the recombination efficiency of each MAGE cycle was dropped by less than 2% when inserting more than 20 bp in sequence. Thus, direct replacement of multiple promoters in genome was laborious and time-consuming. Selecting more recombination-competent cells, co-selection MAGE (CoS-MAGE) overcame this limitation [43]. Selection steps using selection markers dispersed throughout the genome increased the insertion efficiency up to 25% at the region near the selection marker. Using advanced selection strategy, they combinatorially replaced 12 native promoters on the genome with T7 promoter. After all the efforts to increase recombination efficiency, indirect selection methods were not sufficient for recursive recombination to introduce mutations into the genome. Therefore, Ronda et al. improved the efficiency of MAGE-based recombination by implementing CRISPR/Cas9-based selection [44]. While performing the CRISPR-optimized MAGE-recombineering, cells with altered target DNA sequences could survive only.

Despite the high increase in recombineering efficiency, the CRISPR/Cas9-based selection methods could target single loci at each MAGE cycle. High-throughput oligo pool synthesis and automated recombineering enabled incorporating mutations to multiple genomic regions. Garst et al. developed a method that could not only generate genetic perturbation efficiently but also identify the modified region by barcode in the genome-scale [45]. CRISPR-enabled trackable genome engineering (CREATE) was based on the design of a synthetic cassette composing both homology arms for recombination and cognate gRNA for selection. After the CREATE cycle proceeds, the mutations can be traced by sequencing the plasmid from enriched population or cell. This strategy was successfully demonstrated in screening for mutations that confer acetate or furfural tolerance from CREATE libraries. Liu et al. demonstrated iterative use of CREATE system by adding the gRNA plasmid curing step, iCREATE, to elicit combinatorial mutations that permit epistasis [46]. Repeated CREATE cycles using RBS library or pre-determined 28 genes library enhanced hydrolysate tolerance.

Transformation step introducing genetic variants created by in vitro methods into cells is regarded as a limiting step of directed evolution. In vivo continuous generation and accumulation of mutations can accelerate directed evolution. To accomplish this, in vivo continuous evolution (ICE) was demonstrated by Crook et al. (Fig. 1f) [6]. ICE is a retro element-based method which could generate mutations with error-prone nature and be re-integrated into a stable genetic element. Several parameters such as CARGO expression level, host factor, transposase induction conditions, and overexpression of initiator tRNA were investigated to elevate transposition. Compared with error-prone PCR, key factors such as subculture frequency, growth rate, and final OD_600_ of the resulting clones were higher, indicating outperformance of ICE to mutagenesis. Although this system can be implemented in other yeast strains, the absence of transposition system that enables re-integration into originated locus makes it difficult to be performed in *E. coli*. Simon et al. demonstrated a similar system *in E. coli* [47]. Combined use of retroelement of *E. coli* and error-prone T7 RNA polymerase allowed a continuous mutagenesis in *E. coli*. However, relatively low efficiency of re-integration and narrow region that was targeted to be overwritten were remained to be solved.

In vivo random mutagenesis was developed using error-prone DNA polymerase I (Pol I) that is responsible for the replication of ColE1 origin of replication [4]. Although Pol I is involved in lagging strand synthesis during replication of chromosome, it can also initiate replication from the ColE1 origin. Therefore, mutations would be loaded in sequences on a ColE1-containing plasmid by error-prone Pol I during plasmid replication. Mutations occurred up to 3 kb away from the origin, but the rate decreased after 650 bp. This system also showed biased substitution of bases.

The mutagenesis system that is highly error prone during replication of the entire plasmid but for chromosome was developed in yeast. Ravikumar et al. applied the orthogonal plasmid replication protein–DNA pair (p1-TPDNAP1) from pGKL1/2 which is the cytoplasmic plasmid system of *Kluyveromyces lactis* to yeast [11]. They modified p1 plasmid to introduce gene of interest and increase error rate of the plasmid using cognate DNAP (TP-DNAP1) variants without increasing the genomic mutation rate during replication. Arzumanyan et al. reported a development of extrachromosomal error-prone replication system using both of pGKL1-TP-DNAP1 and pGKL2-TP-DNAP2. This method may be applied to directed evolution of enzymes with different error rate in a cell [2]. Recently, Ravikrmar advanced the previously reported error-prone orthogonal plasmid system for high error rates which exceed mutation-induced extinction threshold (4.72 × 10^−6^ s.b.p. for yeast) [48]. The advanced OrthoRep, an orthogonal DNA polymerase–plasmid pair replicated in error-prone manner, consisted of TP-DNAP1 variant which was screened for high error rate and showed around 100,000-fold faster error rate than chromosome. In addition, it was demonstrated that copy number of p1 could be controlled by expressing replication-deficient TP-DNAP1 variants as a competitor. The utility of the highly error-prone and stable mutagenic TP-DNAP1 was demonstrated by evolution of *Plasmodium falciparum* dihydrofolate reductase (PfDHFR) to be resistant to pyrimethamine, an antimalarial drug. High-throughput evolution of PfDHFR, 90 independent replicates, made it possible to trace evolutionary path and complex fitness landscape of drug resistance.

#### Random mutation at the targeted region

Introducing mutations at the designed positions such as promoters, coding regions or regulatory noncoding RNA sequences could be an effective method to expand phenotypic space. Although in vitro assembly of genetic fragments is generally used for site-specific saturation mutagenesis, the library size is limited by transformation efficiency. An iterative use of MAGE-derived techniques can generate saturated mutations into specific loci; it is still limited by transformation and recombineering efficiency.

Thus, several synthetic biology tools have been developed to overcome the limitations of in vitro mutagenesis and to efficiently generate mutations on the intended regions. Recently developed three synthetic tools are introduced in this section. In these methods, the functional proteins are recruited on the target region depending on the interactions between nucleic acids and proteins. Nishida et al. developed a method that could target activation-induced cytidine deaminase (AID) activity to specific locus in yeast and mammalian cells [49]. The gRNA recruited Target-AID, a complex of nickase Cas9 (nCas9) and AID (PmCDA1), at target sequence, and the linked AID generated mutations on the target position. Depending on the gRNA sequence, Target-AID could recruit any locus without any change in genetic background; thus, it is useful for introducing specific mutations (C to T) in a narrow range.

In addition to development of base-editor, Halperin et al. developed a mutator, EvolvR, which not only targeted user-defined region but also generated more variable sequence changes in a wider range [50]. A fusion protein with an error-prone DNA polymerase (DNAP) and nCas9 was devised, and then nCas9 affinity was modified to enhance mutagenesis (Fig. 1g). The DNAP was also engineered to get a different mutation rate, and additional fusion of thioredoxin-binding domain (TBD) could increase the length of the mutable window. These mutator variants allowed users to tune mutation rate. In addition, DNAP was replaced with a Phi29 polymerase mutant, a processive polymerase with reduced fidelity, to increase the tunable window up to 350 bp from PAM.

Another powerful method, MutaT7, was developed which could generate mutations in targeted region [51]. Comparing with EvolvR, the MutaT7 in which a cytidine deaminase (rApo1) fused to T7 RNAP was recruited targeted region by T7 RNAP and T7 promoter interaction (Fig. 1h). The mutations were loaded at targeted region whenever T7 RNAP processed transcription; so strong T7 promoter could show higher mutation rate. Like Target-AID methods, other DNA modifiers would make it possible to generate more diverse variations at defined regions. In addition, MutaT7 could generate almost all mutations in designed region by the nature of T7 RNAP, from T7 promoter to terminator. Both EvolvR and MutaT7 are based on chimeric mutator that is directed to specific regions by gRNA or T7 promoter, respectively. Therefore, multiplex targeting can be easily achieved by using multiple gRNAs or by positioning the T7 promoter at several positions. Depending on whether mutations are intended in multiple targeted windows or genome scale, one of the three effective mutagenesis techniques can be used for each purpose. EvolvR and MP technologies are especially easy to use because they do not require host engineering.

#### Genome-wide random mutagenesis

Although in vivo mutagenesis has advantages over in vitro methods to enhance random mutagenesis in a genome, it has drawbacks such as low efficiency, uncontrolled mutagenesis, and genomic instability. Traditional in vivo mutagenesis by mutator strain also had these problems [52]. To overcome these huddles, Badran et al. developed a mutagenic system based on the mutagenesis plasmid (MP) carrying mechanism-guided mutator genes (Fig. 1i) [53]. The error-prone DNA polymerase III subunit, DnaQ926, was overexpressed in combination with genes known to conferring replication fidelity such as proofreading, MMR, translesion synthesis, and base selection to alter cellular canonical network. Although DnaQ926 alone showed a high mutation rate, mutagenesis plasmid carrying additional genes, MP6, increased mutation rate 63-fold higher than DnaQ926. The utility of the outperformed mutagenesis plasmid was demonstrated by improving antibiotic resistance of a cell and to evolve T7 RNAP to recognize different promoter. Being adapted with phage-assisted continuous evolution (PACE) [54], MP6 could successfully improve soluble expression of protein and diversify PAM sequences recognized by Cas9 [55, 56].

Advanced DNA synthesis technology enabled writing synthetic chromosome and initiating synthetic yeast genome project, Sc2.0. One of the design principles in this project was to provide controllable genetic flexibility. In addition to replace amber stop codon to TAA stop codon, loxPsym sequences were integrated into multiple sites in synthetic chromosome and used for scrambling chromosome under induction [57]. This synthetic chromosome was named as synIXR and allows the SCRaMbLE, synthetic chromosome rearrangement and modification by loxP-mediated evolution. Induction of Cre recombinase in synIXR perturbed yeast phenotype by causing random inversions and deletions in chromosome (Fig. 1j). Many studies demonstrating the applicability of SCRaMbLE to generate phenotypic diversity in yeast were reported [24, 58–64]. Other than entire chromosome, a synthetic pathway can be also adopted for SCRaMbLE. There were studies that have used SCRaMbLE for tuning expression level or pathway structure in combination [63, 64]. By positioning loxPsym sites between genes or upstream and downstream of the promoters, a plasmid could be subjected for SCRaMbLE and resulted in pathway variants. In addition to the techniques to generate phenotypic diversity, a method was developed that efficiently identify SCRaMbLEd variants. Luo et al. demonstrated ReSCuES (reporter of SCRaMbLEd cells using efficient selection) that used two auxotrophic markers; one of them was functional only before SCRaMbLE, and the other was only functional after SCRaMbLE to efficiently distinguish whether Cre was actively expressed or not [62]. The MP and SCRaMbLE methods can generate a genome-wide random mutation, and the mutagenesis can be occurred by induction since the use of an inducible promotor. Mutations accumulate as the duration of mutator expression increases.

Next, a technique to dynamically control a mutator expression was developed. Equipped with appropriate sensor, the mutator expression was stopped when the cell acquired an improved phenotype such as high chemical production. In feedback-regulated evolution of phenotype (FREP), mutation rate was programmed to control the mutagenic gene expression, *mutD5*, for the desired phenotype. Using a sensor recognizing a desirable phenotype such as metabolite production, expression of the mutagenic gene could be stopped at high target molecule concentration, but expression of the mutagenic gene would be induced to accelerate phenotype improvement when cellular concentration of target molecules gets low [65]. This convertible mutagenic state was adopted to evolve cells to resistance in acids [66].

Luan et al. developed Genome Replication Engineering Assisted Continuous Evolution (GREACE) in which mutagenesis was performed with simultaneous selection [67]. They devised in vivo mutagenesis using proofreading element library under selective conditions differing from the traditional mutagenesis where mutagenesis and selection were proceeded sequentially. This strategy was applied to achieve improved tolerance for organic solvents, organic acids, and heat [68]. The adaptive laboratory evolution (ALE) is an effective method to evolve both model and non-model strains when appropriate synthetic tools are not developed. A native xylose fermenting yeast, *Scheffersomyces stipites* was evolved to tolerate ethanol and concentrated hydrolysate. Through the long term of ALE, repetitive culturing in two types of hydrolysates containing ethanol allowed them to screen mutant strains with higher tolerance in hydrolysates with high ethanol concentration [69]. Evolution for cells having tolerance to toxic chemicals or growth inhibition products is relatively straightforward because it is possible to screen based on the growth rate change only. However, for higher production of general chemicals, a method should be able to screen the improved phenotype in high-throughput manner.

### Acquisition of improved phenotypic variant

**Fig. 2:**
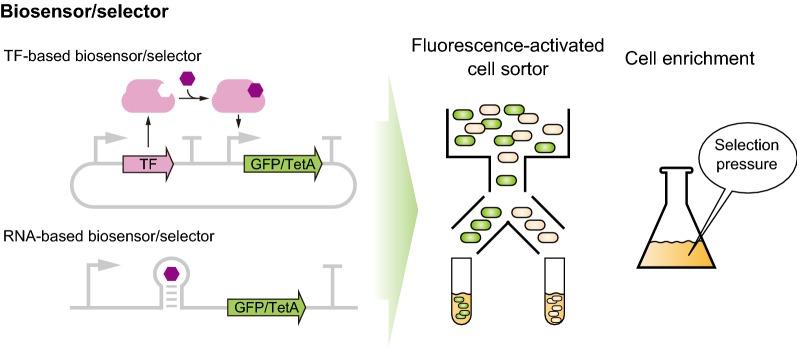
Biosensor/selector construction by assembling sensing module such as TF or riboswitch with reporter. The reporter gene is regulated by a sensing module which detects chemical, different pH, etc. then converts signals detectable in high-throughput such as fluorescence and growth rate under selective pressure

Screening improved strain from the library is another important task in evolutionary engineering. Especially, recently developed techniques that generate vast libraries require high-throughput screening (HTS) methods to efficiently search expanded phenotypic space. In vivo biosensors that detect target chemicals, pH, and temperature in a cell allow HTS. There are two major categories in sensing devices which are protein-based and RNA-based sensor, respectively. Both have been either discovered in nature or synthesized by various methods. They can be assembled with a reporter gene or a selection marker, allowing to convert various phenotypes into signals which can be analyzed in high-throughput manner (Fig. 2).

#### Biosensor-based screening

Lysine-specific biosensor consisting of lysine-specific transcription factor (LysG) and a reporter gene (YFP) was constructed and employed to screen high lysine producers among *Corynebacterium glutamicum* library generated by chemical mutagenesis [70]. With fluorescence-activated cell sorter (FACS) analysis, highly improved mutant strains were screened (Fig. 2). The sequences of the l-lysine synthesis pathway genes and the entire chromosome of the mutant were also analyzed to understand the basic mechanism for improving l-lysine production.

There is less number of direct sensing devices than the number of metabolites of our interests. Therefore, researchers had implemented an additional genetic cassette, helper enzymes, to convert targeted molecules into other chemicals that can be detected by previously developed sensor devices and imaging device [71–75] (Table 1). Rogers et al. circumvented a lack of a sensor to detect 3-hydroxyl propionic acid (3-HP) by introducing a pathway converting 3-HP into acrylate [71]. Through this study, the research group also completed the synthesis pathway from glucose to acrylate for the first time. Nguyen et al. utilized the 3-HP sensor as a 1,3-propanediol (PDO) sensor by introducing enhanced α-ketoglutaric semialdehyde dehydrogenase (KGSADH) that can convert 1,3-PDO into 3-HP [72].

Developing biosensors expands the range of targeted traits that can be acquired. It is easier to create an RNA-based biosensor than to create a TF-based biosensor. Moreover, compared with creating a novel sensor, modifying the dynamic range and targeting binding affinity of existing sensors are not too difficult. pH biosensor that responds to different levels of pH was developed based on the existing pH riboswitches. Then, adaptive evolution was performed under different pHs with genetic cassette harboring pH biosensor [66]. In that genetic cassette, error-prone *dnaQ* and *rfp* were arranged to share a promoter in opposite direction. This promoter was inverted by a site-specific invertase whose expression is controlled by pH riboswitch to allow RFP expression. Thus, the cellular state was programmed the transition from a mutagenic state expressing the mutant DnaQ into a reporting state expressing RFP.

As mentioned above, in vivo biosensor has been applied with a high-throughput instrument to isolate desired phenotype from large library. Fluorescence-activated droplet sorting (FADS) was developed to reduce stress which was applied to cells during sorting by conventional FACS. A tryptophan biosensor was constructed based on the tryptophan riboswitch, and the cells transformed with biosensor were subjected to generate library. Using FADS, pico-litter-sized droplets containing each of tryptophan-producing cell variants were generated and sorted by fluorescence [76]. An alternative method was developed to screen metabolite producer when the targeted metabolite is easily secreted out of the cells. In this case, the intracellular metabolite concentration could not reflect production efficiency of the cell. Thus, a chemical producer and a sensor cell were encapsulated in nano-liter reactors (nLRs) and these nLRs, the alginate beads, were screened using complex object parametric analyzer and sorter (COPAS) device [77]. Because there is no need to change biosensor components such as plasmid, promoter, RBS, or reporter gene, whole-cell biosensors can be easily adopted to screen a library based on a different species. Similarly, a naringenin sensor cell could be used for monitoring production through co-culturing cells with producing cells [78]. In addition to be used for screening of metabolite producing cells, fluorescent reporter can also be used for visualizing other protein expression level. By in vitro translation of *gfp* and target genes in the same tube, expression level of target gene could be inversely correlated with the GFP fluorescence level since the *gfp* and target genes share the limited amount of resources for translation [79].

**Table 1:** List of helper enzymes

Target product	Regulatory protein/detection method	Helper enzyme	Detectable chemical	References
3-HP	PrpR (*Escherichia coli*)	Pcs, PrpC	2-Methylcitrate	[71]
3-HP	AcuR (*Rhodobacter sphaeroides*)	*pcs* mutant, *ach*	Acrylate	[71]
1,3-PDO	MmsR (*Pseudomonas denitrificans*)	KgsA mutant	3-HP	[72]
ω-Amino fatty acids	NitR (*Alcaligenes faecalis*)	Cyclase	Lactam	[73]
Tyrosine	Dark pigment	MelA (*Rhizobium etli*)	Melanin	[74]
Malonyl-CoA	Colorimetric indicator	RppA (*Streptomyces griseus*)	FLAVIOLIN	[75]

#### Selection

Tetracycline resistance gene (*tetA*) has been generally used as selection marker in biosensor construction (Fig. 2). TetA can be used for dual-selection system because tetracycline efflux pump (TetA) not only makes cells resistant to tetracycline, but also makes cells sensitive to nickel. There are successful cases that use tetA-based selection device to acquire novel RNA molecules and improved strains. TetA gene was assembled in biosensors with lysine-riboswitch, naringenin-responsive TF, glucaric acid-responsive TF, and 3-HP-responsive TF to construct a selection device for the improvement of production of each corresponding biochemical by evolutionary engineering of strains [80–83]. Once the cells are equipped with the selection device, improved strains could be simply screened by enrichment culture because they had growth advantage under selection pressure (Fig. 2). Recently, a 3-HP-responsive selection device was constructed and used for evolutionary engineering of aldehyde dehydrogenase (ALDH) [82]. The structure prediction-based ALDH library was subjected to enrichment culture with selection device in that tetA gene expression was controlled by 3-HP-responsive TF. An improved variant exhibited 2.79-fold higher specific activity and the cell harboring this mutant showed higher 3-HP production titer. This easy and fast isolation step can accelerate evolution of metabolic enzyme without expensive equipment. Although many other antibiotic resistance genes can be used as selection marker, the dual-selection strategy by a single selection marker and a resistance mechanism without antibiotics degradation make *tetA* still being an attractive part.

Leavitt et al. demonstrated a strategy surrogating desired phenotype to other phenotype for which a biosensor already existed [84]. They firstly aimed to improve aromatic amino acid (AAA) production using AAA biosensor, and then flux was redirected to muconic acid based on the AAA high-producing variant. This method can be applied to such cases that the sensors responding to the final products are difficult to be developed.

Reduced production efficiency has usually been observed during the long-term cultivation. This is caused by stochastic gene expression that generates phenotypic perturbation without genetic alterations. The synthetic addiction strategy keeps populations of highly producing cells through arresting growth of nonproducing cells [85]. To synthetically addict cells to target product, essential genes of producer are controlled under the product sensing device. If productivity decreases by some reasons such as an epigenetic expression change or an evolutionary gained mutation for toxic product, growth of the cells equipped with addiction device will be arrested. The synthetic addiction of cells to mevalonic acid (MVA) was accomplished using MVA sensing device and essential genes, *folP* and *glmM.*

#### Biomolecule enrichment

The concept of continuously evolving biomolecules in vitro was demonstrated by Wright and Joyce [86]. They targeted RNA ribozyme functioning as RNA ligase to evolve. Ribozyme variants catalyzed ligation with a piece of RNA substrate fused to T7 RNA polymerase promoter. The ligation reaction was beneficial to amplify itself when T7 RNAP was added; therefore, the evolved ligase ribozymes were dominated in the reaction mixture. However, it was difficult to generalize in vitro evolution strategy. The target activity was limited in *cis* manner since all variants were in the same pool. Compartmentalizing each genotype variant is required to analyze genotype–phenotype relationship in high-throughput manner. Tawfik and Griffiths et al. [87] developed a compartmentalization technique which allowed reproduction of genotype–phenotype linkage in vitro. This encapsulation technique was adopted to compartmentalize self-replication (CSR) [88] in which a highly active DNAP can generate more copies of DNA in the emulsion without cross-reaction. They screened thermo-tolerant variant at higher temperature of denaturing stage of PCR and screened heparin (inhibitor for many of polymerases)-tolerant variant through PCR under heparin containing mixture.

**Fig. 3:**
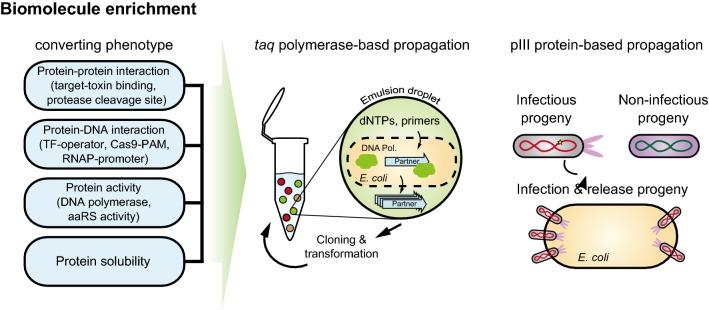
DNAs harboring beneficial mutations are propagated by compartmentalization technique and PACE. Genetic cassettes convert diverse protein functions such as protein–protein binding, protein–DNA interaction, protein specific activity, and protein solubility to changes in the expression levels of *taq*-polymerase or of phage infection protein. In the *taq*-polymerase case, the amount of amplified target product depends on taq-polymerase content which is expressed in a cell. Each cell containing a plasmid carrying target DNA and *taq*-polymerase is encapsulated together with PCR mixture (PCR buffer, dNTPs, primers). During emulsion PCR, cells are disrupted and expose plasmid as template and expressed taq-polymerase. In M13 protein III (pIII) case, expression of pIII is regulated. When beneficial mutations are occurred and increasing pIII expression, phage carrying these mutations can generate more progeny

Ellefson et al. expanded evolution targets to protein–DNA interaction (orthogonal T7 RNAP) and a pair of tRNA synthetase/suppressor tRNAs [89]. They demonstrated that compartmentalized partnered replication (CPR) based on the genetic circuit which links desired phenotype to *taq*-polymerase expression could evolve RNA polymerase and orthogonal translation system efficiently (Fig. 3) [90]. Recently, using a regulatory circuit as a partner, iterative CPR was performed to evolve transcription regulator, Trp repressor, to bind noncanonical effector molecule or to recognize novel operators [91].

Coupling enrichment to the function of interest makes continuous evolution easy and fast. Esvelt et al. demonstrated phage-assisted continuous evolution (PACE) in which evolved phenotypes were linked to generate more offspring [54]. In PACE, effective mutations increased pIII production in host cells and resulted in more infectious phage progeny (Fig. 3). They examined the coupling of infectious phage generation with diverse protein functions such as polymerase activity, protein–peptide binding, and recombinase activity. Constant flow system effectively washed out nonfunctional molecules. Fast phage life-cycle accelerated the evolution rate and resulted in 12 evolution rounds per day. It could last 1 day without human intervention.

**Fig. 4:**
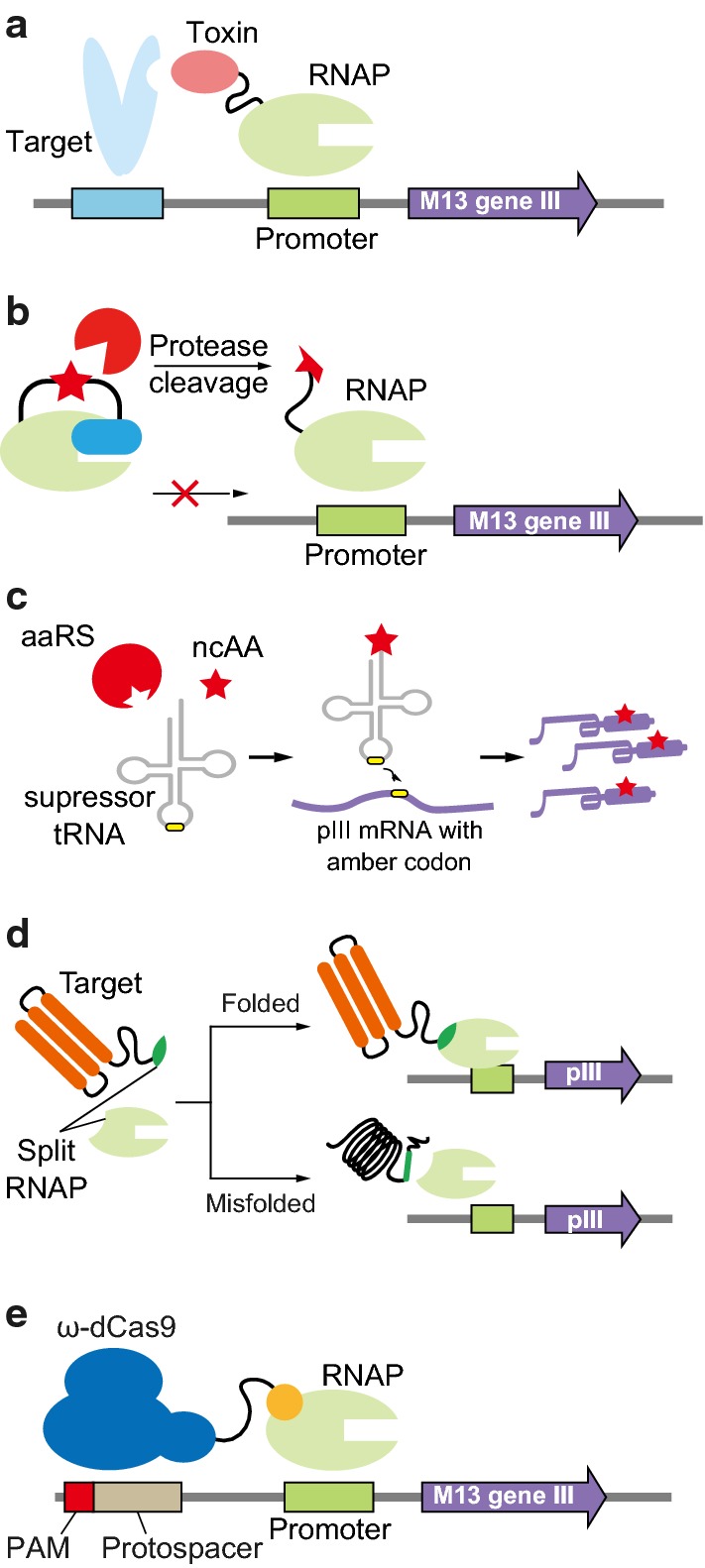
Genetic cassette designs being applied for evolution of protein functions with PACE. **a** Bt toxin evolution to targeting new receptor. **b** TEV protease evolution to target new cleavage site. **c** aaRS evolution to get specificity with ncAA and suppressor tRNA. **d** Eukaryotic protein evolution to improve solubility in *E. coli*. **e** Cas9 evolution to recognize new PAM

PACE can rapidly evolve diverse protein functions, if a genetic circuit is devised, which links pIII synthesis with desired functions. Badran et al. screened improved Cyr1Ac, a wildly used insecticidal protein, also known as Bt toxin, with genetic circuit by coupling protein–protein interaction to pIII synthesis [92]. They designed that pIII could be produced when evolved rpoZ-Bt toxin binds to TnCAD, an insect cell membrane cadherin-like receptor from cabbage looper (*Trichoplusia ni*) (Fig. 4a). The value of proteases that recognize specific targets has been increased in both commercial enzyme and therapeutics industry. Packer et al. evolved TEV protease to cleave a completely new target amino acid sequence [93]. T7 RNAP was fused with T7 lysozyme through a linker that could be only cleaved by evolved proteases (Fig. 4b). PACE allowed to find a novel TEV-derived protease which could cleave human IL-23 without loss of activity to the consensus substrate. Meanwhile, these results indicated the need for negative selection strategy to eliminate activity to unintentional substrates. Bryson et al. also used PACE to evolve orthogonal aminoacyl-tRNA for the incorporation of noncanonical amino acids (ncAAs) [94]. They performed PACE using T7 RNAP or pIII containing amber codons at a position, so that premature termination can be occurred if ncAAs are not incorporated by evolved aaRS/tRNA (Fig. 4c). In addition, negative selection step utilizing pIII-neg which was non-infectious pIII expressed without ncAAs, reduced unintentional function such as an activity on endogenous amino acids. Moreover, PACE employed to modify a split RNAP system to rewire assembly of split parts into proximity-dependent manner. In order to expand the uses of split RNAP in biosensor, Pu et al. fused leucine zipper peptide into each of RNAP parts, and N terminus of RNAP was subjected to mutagenesis [95]. The mutated RNAP was selected only when assembly of RNAP was assisted by peptide–peptide interaction and named as activity-responsive (AR) RNAP system. The applicability of AR system was validated through developing both light and small-molecule biosensors by replacing the protein–protein interaction domain with domains conferring light and rapamycin responsive dimerization, respectively.

PACE was also applied to improve protein–DNA interaction in CRISPR system. To expand PAM sequence to various target positions, Cas9 protein was subjected into evolution. The gene cassette was constructed for pIII expression only when dCas9 binds to a protospacer with a modified PAM sequence [55]. Recognition of new PAM by dCas9-rpoZ fusion protein triggers transcription of pIII by *E. coli* RNAP (Fig. 4e). Through PACE, xCas9 having enhanced binding affinity, broadened PAM sequences, and reduced off-target activity was screened. The PAM specificity of Cas9 has been regarded as huddle in CRISPR assisting recombineering or genome editing applications. Therefore, xCas9 will be adopted for various applications utilizing the CRISPR system.

The expression of soluble proteins is essential for the recombinant protein production and application. Thus, there have been various strategies to improve protein solubility. The traditional methods optimizing growth conditions such as temperature, medium, inducer concentration, induction time, and co-expression of molecular chaperones as well as fusion of folding tag are still applied for enhance protein solubility [96, 97]. In spite of many studies, the difficulty in protein folding is not easy to be solved just using environmental optimization, because the folding is largely affected by the properties of proteins such as the amino acid sequences. The directed evolution may improve protein solubility, but since the size of the library is so large, an efficient screening method should be followed. Utilizing folding reporters was one of the strategies to screen protein variants in high-throughput manner. However, fusion of large domains sometimes causes loss of function of partner protein. Split RNA polymerase (T7 RNA polymerase small residue and rpoZ) was used as a reporter of soluble expression [56]. By fusion of a target protein with the small fragment of split RNAP (T7n and rpoZ), soluble expression variants triggered pIII expression by exposing the RNAP domain to bind with another fragment (Fig. 4d). They also used split pIII system to develop AND gate to allocate each function of interest. Protein binding activity and soluble expression were linked to the expression of each pIII part.

## Conclusions

Devising gene cassettes or genetic circuits that perform specific functions has long been performed in synthetic biology field. Synthetic biology tools have been developed to generate mutations in the cell with different mutation rate, mutation range (specific regions or entire genome), multiplexity, and whether the altered phenotypes are permanent or removable. The power of these tools was usually examined through improving specific phenotypes such as production of colored chemicals or tolerance. However, industrially interesting chemicals are generally indistinguishable chemicals or provide no growth benefit to producers, and thus the step of acquiring improved variants from a huge library is considered as a major limitation.

Thus, the development of synthetic systems that convert specific chemical concentration or desired protein functions into selectable phenotype is another key for successful evolutionary engineering. For example, the use of biosensor that links the metabolite concentration to detectable phenotype such as fluorescence and resistance to selection pressure was exemplified for lysine, naringenin, glucaric acid, mevalonic acid, 3-HP, and so on. Therefore, discovering natural regulators and developing new regulators using synthetic parts will empower the biosensor-based screening/selection step. Moreover, well-designed accessory plasmids for PACE have expanded target functions to various properties. Multiple traits can also be satisfied by complex logic gates.

Altogether, the advance in synthetic biology provides efficient tools for the two main steps of evolutionary engineering: genetic perturbation and acquisition of improved phenotype. Now, it is pushed to identify effective changes from randomly generated mutations. Analyzing the consequences of mutations on cellular/protein functions is an additional step for evolutionary engineering. Recently, facilitated omics technology has assisted evolutionary engineering. Using next-generation sequencing, tracking genome-wide multiple mutations is plausible. Moreover, the analysis of accumulated mutation in the evolved populations allows to study evolutionary paths.

The growing interest of bioindustry demands much complex phenotype or use of non-model strain. Therefore, the utility of evolutionary engineering which can optimize cellular phenotype even without intensive biological information will increase. The synthetic genetic tools will accelerate evolution of much diverse phenotypes in various microbes.

## Data Availability

Not applicable.

## References

[1] Alper H, Moxley J, Nevoigt E, Fink GR, Stephanopoulos G (2006). Engineering yeast transcription machinery for improved ethanol tolerance and production. Science.

[2] Arzumanyan GA, Gabriel KN, Ravikumar A, Javanpour AA, Liu CC (2018). Mutually orthogonal DNA replication systems in vivo. ACS Synth Biol..

[3] Bikard D, Jiang W, Samai P, Hochschild A, Zhang F, Marraffini LA (2013). Programmable repression and activation of bacterial gene expression using an engineered CRISPR-Cas system. Nucleic Acids Res.

[4] Camps M, Naukkarinen J, Johnson BP, Loeb LA (2003). Targeted gene evolution in *Escherichia coli* using a highly error-prone DNA polymerase I. Proc Natl Acad Sci USA.

[5] Chen T, Wang J, Zeng L, Li R, Li J, Chen Y, Lin Z (2012). Significant rewiring of the transcriptome and proteome of an *Escherichia coli* strain harboring a tailored exogenous global regulator IrrE. PLoS ONE.

[6] Crook N, Abatemarco J, Sun J, Wagner JM, Schmitz A, Alper HS (2016). In vivo continuous evolution of genes and pathways in yeast. Nat Commun..

[7] Dong C, Fontana J, Patel A, Carothers JM, Zalatan JG (2018). Synthetic CRISPR-Cas gene activators for transcriptional reprogramming in bacteria. Nat Commun.

[8] Na D, Yoo SM, Chung H, Park H, Park JH, Lee SY (2013). Metabolic engineering of *Escherichia coli* using synthetic small regulatory RNAs. Nat Biotechnol.

[9] Park K-S, Lee D-K, Lee H, Lee Y, Jang Y-S, Kim YH, Yang H-Y, Lee S-I, Seol W, Kim J-S (2003). Phenotypic alteration of eukaryotic cells using randomized libraries of artificial transcription factors. Nat Biotechnol..

[10] Qi LS, Larson MH, Gilbert LA, Doudna JA, Weissman JS, Arkin AP, Lim WA (2013). Repurposing CRISPR as an RNA-guided platform for sequence-specific control of gene expression. Cell.

[11] Ravikumar A, Arrieta A, Liu CC (2014). An orthogonal DNA replication system in yeast. Nat Chem Biol.

[12] Wang HH, Isaacs FJ, Carr PA, Sun ZZ, Xu G, Forest CR, Church GM (2009). Programming cells by multiplex genome engineering and accelerated evolution. Nature.

[13] Seong YJ, Park H, Yang J, Kim SJ, Choi W, Kim KH, Park YC (2017). Expression of a mutated *SPT15* gene in *Saccharomyces cerevisiae* enhances both cell growth and ethanol production in microaerobic batch, fed-batch, and simultaneous saccharification and fermentations. Appl Microbiol Biotechnol.

[14] Liu H, Liu K, Yan M, Xu L, Ouyang P (2011). gTME for improved adaptation of *Saccharomyces cerevisiae* to corn cob acid hydrolysate. Appl Biochem Biotechnol.

[15] Wadhwa M, Srinivasan S, Bachhawat AK, Venkatesh KV (2018). Role of phosphate limitation and pyruvate decarboxylase in rewiring of the metabolic network for increasing flux towards isoprenoid pathway in a TATA binding protein mutant of *Saccharomyces cerevisiae*. Microb Cell Fact.

[16] Wadhwa M, Bachhawat AK (2016). A genetic screen for increasing metabolic flux in the isoprenoid pathway of *Saccharomyces cerevisiae*: isolation of SPT15 mutants using the screen. Metab Eng Commun.

[17] Wang M, Liu G-N, Liu H, Zhang L, Li B-Z, Li X, Liu D, Yuan Y-J (2018). Engineering global transcription to tune lipophilic properties in *Yarrowia lipolytica*. Biotechnol Biofuels.

[18] Zhang F, Qian X, Si H, Xu G, Han R, Ni Y (2015). Significantly improved solvent tolerance of *Escherichia coli* by global transcription machinery engineering. Microb Cell Fact.

[19] Chen T, Wang J, Yang R, Li J, Lin M, Lin Z (2011). Laboratory-evolved mutants of an exogenous global regulator, IrrE from *Deinococcus radiodurans*, enhance stress tolerances of *Escherichia coli*. PLoS ONE.

[20] Appukuttan D, Singh H, Park SH, Jung JH, Jeong S, Seo HS, Choi YJ, Lim S (2016). Engineering synthetic multistress tolerance in *Escherichia coli* by using a deinococcal response regulator, DR1558. Appl Environ Microbiol.

[21] Blazeck J, Hill A, Liu L, Knight R, Miller J, Pan A, Otoupal P, Alper HS (2014). Harnessing *Yarrowia lipolytica* lipogenesis to create a platform for lipid and biofuel production. Nat Commun.

[22] Deaner M, Alper HS (2017). Systematic testing of enzyme perturbation sensitivities via graded dCas9 modulation in *Saccharomyces cerevisiae*. Metab Eng.

[23] Deaner M, Mejia J, Alper HS (2017). Enabling graded and large-scale multiplex of desired genes using a dual-mode dCas9 activator in *Saccharomyces cerevisiae*. ACS Synth Biol.

[24] Jia B, Wu Y, Li B-Z, Mitchell LA, Liu H, Pan S, Wang J, Zhang H-R, Jia N, Li B (2018). Precise control of SCRaMbLE in synthetic haploid and diploid yeast. Nat Commun..

[25] Wu Y, Chen T, Liu Y, Lv X, Li J, Du G, Ledesma-Amaro R, Liu L (2018). CRISPRi allows optimal temporal control of *N*-acetylglucosamine bioproduction by a dynamic coordination of glucose and xylose metabolism in *Bacillus subtilis*. Metab Eng.

[26] Wang T, Guan C, Guo J, Liu B, Wu Y, Xie Z, Zhang C, Xing X-H (2018). Pooled CRISPR interference screening enables genome-scale functional genomics study in bacteria with superior performance. Nat Commun.

[27] Cress BF, Toparlak OD, Guleria S, Lebovich M, Stieglitz JT, Englaender JA, Jones JA, Linhardt RJ, Koffas MA (2015). CRISPathBrick: modular combinatorial assembly of type II-A CRISPR arrays for dCas9-mediated multiplex transcriptional repression in *E. coli*. ACS Synth Biol..

[28] Deaner M, Holzman A, Alper HS (2018). Modular ligation extension of guide RNA operons (LEGO) for multiplexed dCas9 regulation of metabolic pathways in *Saccharomyces cerevisiae*. Biotechnol J.

[29] Zuckermann M, Hlevnjak M, Yazdanparast H, Zapatka M, Jones DTW, Lichter P, Gronych J (2018). A novel cloning strategy for one-step assembly of multiplex CRISPR vectors. Sci Rep..

[30] Salis HM, Mirsky EA, Voigt CA (2009). Automated design of synthetic ribosome binding sites to control protein expression. Nat Biotechnol.

[31] Seo SW, Yang JS, Kim I, Yang J, Min BE, Kim S, Jung GY (2013). Predictive design of mRNA translation initiation region to control prokaryotic translation efficiency. Metab Eng.

[32] Farasat I, Kushwaha M, Collens J, Easterbrook M, Guido M, Salis HM (2014). Efficient search, mapping, and optimization of multi-protein genetic systems in diverse bacteria. Mol Syst Biol.

[33] Seo SW, Yang J-S, Cho H-S, Yang J, Kim SC, Park JM, Kim S, Jung GY (2014). Predictive combinatorial design of mRNA translation initiation regions for systematic optimization of gene expression levels. Sci Rep..

[34] Jeschek M, Gerngross D, Panke S (2016). Rationally reduced libraries for combinatorial pathway optimization minimizing experimental effort. Nat Commun..

[35] Costantino N, Court DL (2003). Enhanced levels of lambda red-mediated recombinants in mismatch repair mutants. Proc Natl Acad Sci USA.

[36] Wang HH, Xu G, Vonner AJ, Church G (2011). Modified bases enable high-efficiency oligonucleotide-mediated allelic replacement via mismatch repair evasion. Nucleic Acids Res.

[37] Oesterle S, Gerngross D, Schmitt S, Roberts TM, Panke S (2017). Efficient engineering of chromosomal ribosome binding site libraries in mismatch repair proficient *Escherichia coli*. Sci Rep..

[38] Noh M, Yoo SM, Kim WJ, Lee SY (2017). Gene expression knockdown by modulating synthetic small RNA expression in *Escherichia coli*. Cell Syst..

[39] Ghodasara A, Voigt CA (2017). Balancing gene expression without library construction via a reusable sRNA pool. Nucleic Acids Res.

[40] Fried SD, Schmied WH, Uttamapinant C, Chin JW (2015). Ribosome subunit stapling for orthogonal translation in *E. coli*. Angew Chem Int Ed.

[41] Orelle C, Carlson ED, Szal T, Florin T, Jewett MC, Mankin AS (2015). Protein synthesis by ribosomes with tethered subunits. Nature.

[42] Wong TS, Arnold FH, Schwaneberg U (2004). Laboratory evolution of cytochrome p450 BM-3 monooxygenase for organic cosolvents. Biotechnol Bioeng.

[43] Wang HH, Kim H, Cong L, Jeong J, Bang D, Church GM (2012). Genome-scale promoter engineering by coselection MAGE. Nat Methods.

[44] Ronda C, Pedersen LE, Sommer MOA, Nielsen AT (2016). CRMAGE: CRISPR optimized MAGE recombineering. Sci Rep..

[45] Garst AD, Bassalo MC, Pines G, Lynch SA, Halweg-Edwards AL, Liu R, Liang L, Wang Z, Zeitoun R, Alexander WG (2016). Genome-wide mapping of mutations at single-nucleotide resolution for protein, metabolic and genome engineering. Nat Biotechnol.

[46] Liu R, Liang L, Garst AD, Choudhury A, i Nogué VS, Beckham GT, Gill RT (2018). Directed combinatorial mutagenesis of *Escherichia coli* for complex phenotype engineering. Metab Eng..

[47] Simon AJ, Morrow BR, Ellington AD (2018). Retroelement-based genome editing and evolution. ACS Synth Biol.

[48] Ravikumar A, Arzumanyan GA, Obadi MKA, Javanpour AA, Liu CC (2018). Scalable, continuous evolution of genes at mutation rates above genomic error thresholds. Cell.

[49] Nishida K, Arazoe T, Yachie N, Banno S, Kakimoto M, Tabata M, Mochizuki M, Miyabe A, Araki M, Hara KY (2016). Targeted nucleotide editing using hybrid prokaryotic and vertebrate adaptive immune systems. Science..

[50] Halperin SO, Tou CJ, Wong EB, Modavi C, Schaffer DV, Dueber JE (2018). CRISPR-guided DNA polymerases enable diversification of all nucleotides in a tunable window. Nature.

[51] Moore CL, Papa LJ, Shoulders MD (2018). A processive protein chimera introduces mutations across defined DNA regions in vivo. J Am Chem Soc.

[52] Greener A, Callahan M, Jerpseth B (1997). An efficient random mutagenesis technique using an *E. coli* mutator strain. Mol Biotechnol.

[53] Badran AH, Liu DR (2015). Development of potent in vivo mutagenesis plasmids with broad mutational spectra. Nat Commun..

[54] Esvelt KM, Carlson JC, Liu DR (2011). A system for the continuous directed evolution of biomolecules. Nature.

[55] Hu JH, Miller SM, Geurts MH, Tang W, Chen L, Sun N, Zeina CM, Gao X, Rees HA, Lin Z (2018). Evolved Cas9 variants with broad PAM compatibility and high DNA specificity. Nature.

[56] Wang T, Badran AH, Huang TP, Liu DR (2018). Continuous directed evolution of proteins with improved soluble expression. Nat Chem Biol.

[57] Dymond JS, Richardson SM, Coombes CE, Babatz T, Muller H, Annaluru N, Blake WJ, Schwerzmann JW, Dai J, Lindstrom DL (2011). Synthetic chromosome arms function in yeast and generate phenotypic diversity by design. Nature.

[58] Wang J, Xie Z-X, Ma Y, Chen X-R, Huang Y-Q, He B, Bin J, Li B-Z, Yuan Y-J (2018). Ring synthetic chromosome V SCRaMbLE. Nat Commun..

[59] Blount BA, Gowers GOF, Ho JCH, Ledesma-Amaro R, Jovicevic D, McKiernan RM, Xie ZX, Li BZ, Yuan YJ, Ellis T (2018). Rapid host strain improvement by in vivo rearrangement of a synthetic yeast chromosome. Nat Commun..

[60] Hochrein L, Mitchell LA, Schulz K, Messerschmidt K, Mueller-Roeber B (2018). L-SCRaMbLE as a tool for light-controlled Cre-mediated recombination in yeast. Nat Commun..

[61] Shen MJ, Wu Y, Yang K, Li Y, Xu H, Zhang H, Li B-Z, Li X, Xiao W-H, Zhou X (2018). Heterozygous diploid and interspecies SCRaMbLEing. Nat Commun..

[62] Luo Z, Wang L, Wang Y, Zhang W, Guo Y, Shen Y, Jiang L, Wu Q, Zhang C, Cai Y (2018). Identifying and characterizing SCRaMbLEd synthetic yeast using ReSCuES. Nat Commun..

[63] Wu Y, Zhu RY, Mitchell LA, Ma L, Liu R, Zhao M, Jia B, Xu H, Li YX, Yang ZM (2018). In vitro DNA SCRaMbLE. Nat Commun.

[64] Liu W, Luo Z, Wang Y, Pham NT, Tuck L, Perez-Pi I, Liu L, Shen Y, French C, Auer M (2018). Rapid pathway prototyping and engineering using in vitro and in vivo synthetic genome SCRaMbLE-in methods. Nat Commun..

[65] Chou HH, Keasling JD (2013). Programming adaptive control to evolve increased metabolite production. Nat Commun..

[66] Pham HL, Wong A, Chua N, Teo WS, Yew WS, Chang MW (2017). Engineering a riboswitch-based genetic platform for the self-directed evolution of acid-tolerant phenotypes. Nat Commun..

[67] Luan G, Cai Z, Li Y, Ma Y (2013). Genome replication engineering assisted continuous evolution (GREACE) to improve microbial tolerance for biofuels production. Biotechnol Biofuels.

[68] Luan G, Bao G, Lin Z, Li Y, Chen Z, Li Y, Cai Z (2015). Comparative genome analysis of a thermotolerant *Escherichia coli* obtained by genome replication engineering assisted continuous evolution (GREACE) and its parent strain provides new understanding of microbial heat tolerance. New Biotechnol.

[69] Slininger PJ, Shea-Andersh MA, Thompson SR, Dien BS, Kurtzman CP, Balan V, da Costa Sousa L, Uppugundla N, Dale BE, Cotta MA (2015). Evolved strains of *Scheffersomyces stipitis* achieving high ethanol productivity on acid- and base-pretreated biomass hydrolyzate at high solids loading. Biotechnol Biofuels.

[70] Binder S, Schendzielorz G, Stäbler N, Krumbach K, Hoffmann K, Bott M, Eggeling L (2012). A high-throughput approach to identify genomic variants of bacterial metabolite producers at the single-cell level. Genome Biol.

[71] Rogers JK, Church GM (2016). Genetically encoded sensors enable real-time observation of metabolite production. Proc Natl Acad Sci USA.

[72] Nguyen NH, Kim J-R, Park S (2018). Application of transcription factor-based 3-hydroxypropionic acid biosensor. Biotechnol Bioprocess Eng.

[73] Yeom SJ, Kim M, Kwon KK, Fu Y, Rha E, Park SH, Lee H, Kim H, Lee DH, Kim DM (2018). A synthetic microbial biosensor for high-throughput screening of lactam biocatalysts. Nat Commun.

[74] Santos CN, Stephanopoulos G (2008). Melanin-based high-throughput screen for l-tyrosine production in *Escherichia coli*. Appl Environ Microbiol.

[75] Yang D, Kim WJ, Yoo SM, Choi JH, Ha SH, Lee MH, Lee SY (2018). Repurposing type III polyketide synthase as a malonyl-CoA biosensor for metabolic engineering in bacteria. Proc Natl Acad Sci USA.

[76] Jang S, Lee B, Jeong H-H, Jin SH, Jang S, Kim SG, Jung GY, Lee C-S (2016). On-chip analysis, indexing and screening for chemical producing bacteria in a microfluidic static droplet array. Lab Chip.

[77] Meyer A, Pellaux R, Potot S, Becker K, Hohmann HP, Panke S, Held M (2015). Optimization of a whole-cell biocatalyst by employing genetically encoded product sensors inside nanolitre reactors. Nat Chem..

[78] Xiu Y, Jang S, Jones JA, Zill NA, Linhardt RJ, Yuan Q, Jung GY, Koffas MAG (2017). Naringenin-responsive riboswitch-based fluorescent biosensor module for *Escherichia coli* co-cultures. Biotechnol Bioeng.

[79] Park YJ, Lee K-H, Baek MS, Kim D-M (2017). High-throughput engineering of initial coding regions for maximized production of recombinant proteins. Biotechnol Bioprocess Eng.

[80] Yang J, Seo SW, Jang S, Shin SI, Lim CH, Roh TY, Jung GY (2013). Synthetic RNA devices to expedite the evolution of metabolite-producing microbes. Nat Commun..

[81] Raman S, Rogers JK, Taylor ND, Church GM (2014). Evolution-guided optimization of biosynthetic pathways. Proc Natl Acad Sci USA.

[82] Seok JY, Yang J, Choi SJ, Lim HG, Choi UJ, Kim KJ, Park S, Yoo TH, Jung GY (2018). Directed evolution of the 3-hydroxypropionic acid production pathway by engineering aldehyde dehydrogenase using a synthetic selection device. Metab Eng.

[83] Nomura Y, Yokobayashi Y (2014). Dual genetic selection of synthetic riboswitches in *Escherichia coli*. Methods Mol Biol.

[84] Leavitt JM, Wagner JM, Tu CC, Tong A, Liu Y, Alper HS (2017). Biosensor-enabled directed evolution to improve muconic acid production in *Saccharomyces cerevisiae*. Biotechnol J.

[85] Rugbjerg P, Sarup-Lytzen K, Nagy M, Sommer MOA (2018). Synthetic addiction extends the productive life time of engineered *Escherichia coli* populations. Proc Natl Acad Sci USA.

[86] Wright MC, Joyce GF (1997). Continuous in vitro evolution of catalytic function. Science.

[87] Tawfik DS, Griffiths AD (1998). Man-made cell-like compartments for molecular evolution. Nat Biotechnol.

[88] Ghadessy FJ, Ong JL, Holliger P (2001). Directed evolution of polymerase function by compartmentalized self-replication. Proc Natl Acad Sci USA.

[89] Ellefson JW, Meyer AJ, Hughes RA, Cannon JR, Brodbelt JS, Ellington AD (2013). Directed evolution of genetic parts and circuits by compartmentalized partnered replication. Nat Biotechnol.

[90] Maranhao AC, Ellington AD (2017). Evolving orthogonal suppressor tRNAs to incorporate modified amino acids. ACS Synth Biol.

[91] Ellefson JW, Ledbetter MP, Ellington AD (2018). Directed evolution of a synthetic phylogeny of programmable Trp repressors. Nat Chem Biol.

[92] Badran AH, Guzov VM, Huai Q, Kemp MM, Vishwanath P, Kain W, Nance AM, Evdokimov A, Moshiri F, Turner KH (2016). Continuous evolution of *Bacillus thuringiensis* toxins overcomes insect resistance. Nature.

[93] Packer MS, Rees HA, Liu DR (2017). Phage-assisted continuous evolution of proteases with altered substrate specificity. Nat Commun.

[94] Bryson DI, Fan C, Guo LT, Miller C, Soll D, Liu DR (2017). Continuous directed evolution of aminoacyl-tRNA synthetases. Nat Chem Biol.

[95] Pu J, Zinkus-Boltz J, Dickinson BC (2017). Evolution of a split RNA polymerase as a versatile biosensor platform. Nat Chem Biol.

[96] Baneyx F (1999). Recombinant protein expression in *Escherichia coli*. Curr Opin Biotechnol.

[97] Sachsenhauser V, Bardwell JCA (2018). Directed evolution to improve protein folding in vivo. Curr Opin Struct Biol.

